# Partnering With Hospices to Develop, Test, and Implement Aliviado Dementia Care in a 25-Site Pragmatic Trial

**DOI:** 10.1093/geroni/igaa057.2852

**Published:** 2020-12-16

**Authors:** Abraham Brody, Tina Sadarangani, Alycia Bristol, Tessa Jones, Kimberly Convery, Donna McCabe, Aditi Durga, Shih-Yin Lin

**Affiliations:** 1 NYU Rory Meyers College of Nursing, New York, New York, United States; 2 New York University, New York, New York, United States; 3 NYU Rory Meyers College of Nursing, New york, New York, United States; 4 NYU Rory Meyers College of nursing, New York, New York, United States

## Abstract

Almost half of hospice patients currently have a diagnosis of dementia. However, few pragmatic, evidence-based, programs have been developed and tested in this setting overall, and targeting persons living with dementia and their caregivers specifically. Furthermore, limited academic-hospice research partnership exist. We thus undertook efforts to develop and test Aliviado Dementia Care-Hospice Edition, a complex multi-modal quality improvement intervention, as part of the HAS-QOL Trial, a 2-phase, 25 site embedded pragmatic clinical trial, through partnership and engagement with hospice and caregiver stakeholders. We discuss methods for engagement and barriers to implementation. Stakeholders assisted with alignment of the intervention to their needs and how to flexibly implement, substantially strengthening the intervention. They also provided important input regarding how to sustain the intervention through technological, mentorship and communication support mechanisms. Barriers found include the nature of implementing in limited resource settings with multiple competing priorities and little research infrastructure.

